# Case Report: Anterior Cruciate Ligament Calcification in a Patient With Chondrocalcinosis: Micro-Computed Tomography Presentation

**DOI:** 10.3389/fsurg.2021.680234

**Published:** 2021-07-29

**Authors:** Alberto Grassi, Giacomo Dal Fabbro, Milena Fini, Stefano Zaffagnini, Annapaola Parrilli

**Affiliations:** ^1^IRCCS Istituto Ortopedico Rizzoli, 2nd Orthopaedic and Traumatologic Clinic, Bologna, Italy; ^2^IRCCS Istituto Ortopedico Rizzoli, RIT Department, Surgical Sciences and Technologies, Bologna, Italy; ^3^Empa - Swiss Federal Laboratories for Materials Science and Technology, Center for X-ray Analytics, Dübendorf, Switzerland

**Keywords:** knee, anterior cruciate ligament, chondrocalcinosis, calcification, Micro-CT (computed tomography), μCT and 3D reconstruction

## Abstract

In this case report, an incidental postoperative diagnosis of anterior cruciate ligament (ACL) calcification, associated with calcification of posterior cruciate ligament (PCL) and lateral meniscus insertions, was made using micro-computed tomography (μCT) technology in a knee specimen obtained during a total knee replacement (TKR) surgery due to painful tri-compartmental osteoarthritis (OA) with chondrocalcinosis signs at preoperative X-ray. Anterior cruciate ligament calcification is an uncommon finding, and conventional X-ray and MRI are not so helpful in its identification. μCT scan, in contrast, is of interest because it provides highly spatial three-dimensional information with excellent visualization of bones and calcifications. The μCT technology used in this case report allowed us to perform a detailed analysis and a 3-D reconstruction of the calcium pyrophosphate dihydrate (CPPD) crystal deposition about the knee without the need to section the specimens into slice as performed in previous studies. The 3-D model obtained with μCT scan permits to gain more insight into the shape of the calcification within the fibers of the ligamentous structures of the joint.

## Introduction

Calcification of the anterior cruciate ligament (ACL), described first in a cadaveric case by Steinbach et al. (1), is a rare clinical entity, and only few cases have been provided in the literature (2, 3). Although some occurrences were reported, it is still not clear if ACL calcification is related to any previous injury or surgery (4–7). Several risk factors for ACL calcification have been analyzed: Thyroid disorders, diabetes, and PTH disorders have been proposed, without being confirmed so far (8).

Chondrocalcinosis is a common disorder in the elderly (9, 10) characterized by calcium pyrophosphate dihydrate (CPPD) crystal deposition in and around articular tissues (11–13) and represents a risk factor for the development of calcification of ACL (3, 14–17). Conventional X-ray is the most commonly used imaging method for the visualization of intra-articular calcium crystal deposition and allows us to recognize CPPD deposition in fibrocartilage, but it lacks in sensitivity limiting an insight into burden and localization (18). MR imaging is limited in the distinction between calcification of cartilage and tissues from other abnormalities (19). Computed tomography (CT) scan, rather, is of interest because it provides highly spatial three-dimensional (3D) information with excellent visualization of bones and calcifications (11, 17, 20). Due to the resolution of these clinical systems and to the challenge in differentiating CPPD deposition from other lesions (18, 19), 3D reconstruction of the knee joint by both CT and MR is usually limited to the reconstruction of the bone segments and used as tools for improving the planning or the outcomes of the knee surgery (21).

The clinical implications of calcification of ACL and its association with ligament degeneration and development of knee osteoarthritis (OA) are still not fully acknowledged because of the lack of clear studies (3, 15).

The aim of the present case report was to describe the tridimensional distribution pattern of ACL calcification in the context of chondrocalcinosis, using a micro-computed tomography (μCT) technology. The hypothesis was that using this method, it would be possible to investigate the shape and course of calcifications within the ligament fibers and from multiple spatial view orientations.

## Case Description

A human specimen of tibial plateau and femoral intercondylar notch including ACL, posterior cruciate ligament (PCL), and meniscal insertions (lateral meniscus) was obtained from a patient with diagnosis of chondrocalcinosis during the surgery for total knee replacement (TKR) due to painful tri-compartmental knee OA. The specimen was scanned with a μCT system, and a 3D reconstruction of the specimen was built. Informed consent was obtained from the patient included in the case report.

### Patient Characteristics

**Figure 1:**
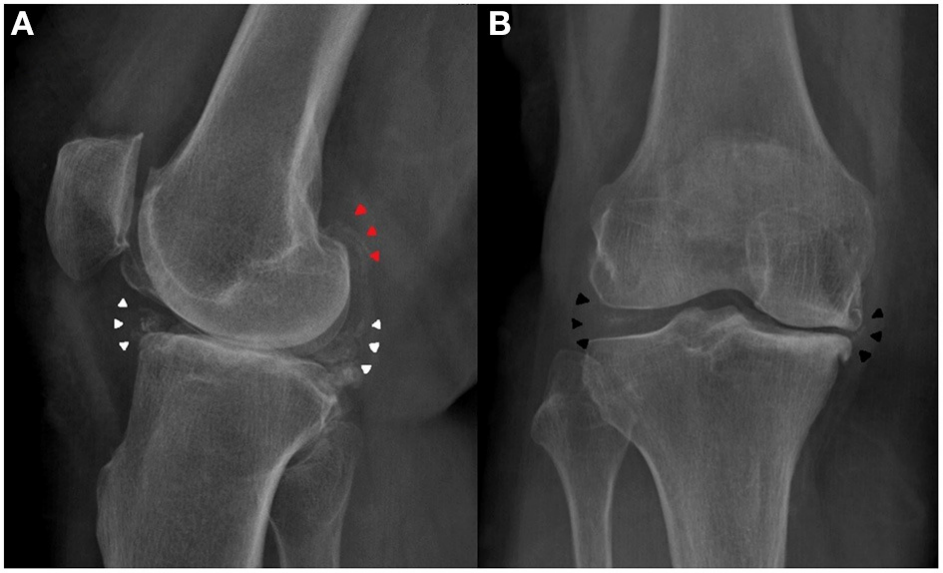
Preoperative knee X-ray of the patient. **(A)** Lateral view showed meniscus (white arrowheads) and gastrocnemius tendon (red arrowheads) calcification. **(B)** Antero-posterior view showed medial and lateral meniscus calcification (black arrowheads).

**Figure 2:**
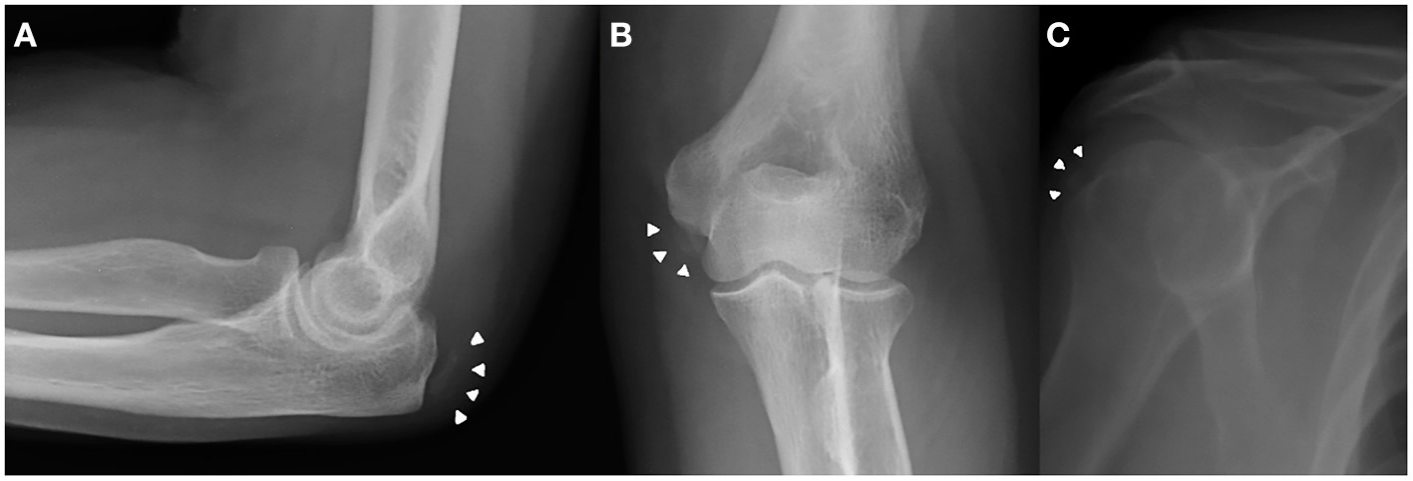
Previous elbow and shoulder X-ray obtained for minor traumatic events revealed the presence of CPPD crystal deposition (white arrowheads) at the level of writs flexors insertion at medial epicondyle **(A,B)** and rotator cuff **(C)** as well.

A 72-year-old housewife presented with a chronic and growing right knee pain for more than 10 years with important functional limitation during normal daily activities. Clinical examination showed a swollen knee with varus malalignment. An acute pain was evoked by palpation of the joint space narrowing. The passive range of motion was restricted from 0° to 90°. The knee were stable under antero-posterior and varus-valgus stress. The X-ray examination showed a tri-compartmental OA of the knee, especially the medial tibia–femoral compartment and patella–femoral compartment. Moreover, X-ray examination showed chondrocalcinosis of the medial and lateral meniscus (Figure 1). Patient denied prior knee trauma or surgery. In her history, she reported a post-thyroidectomy hypothyroidism and osteoporosis. Previous elbow and shoulder radiographs obtained for minor traumatic events revealed the presence of CPPD at the level of rotator cuff and writs flexors insertion at medial epicondyle as well (Figure 2). She was in therapy with cardioaspirin, pantoprazole, ibandronic acid, vitamin D, and levothyroxine. Blood test revealed a normal blood count and normal blood levels of electrolytes, calcium, and phosphorus. Thus, the present patient was indicated for TKR surgery. Following the surgery, a partial weight bearing was set for 2 weeks. Physical therapy and range of motion exercise started 3 days after the surgery. At 3 weeks after the surgery, the patient was cleared to full weight-bearing. At her final follow-up, 1 year postoperatively, the patient was satisfied with the outcomes and still doing well with no complaints of pain, presenting an active range of motion from 0° to 115°.

### Specimen Preparation

During the planned surgery of a mechanically aligned TKR, a standard medial parapatellar approach was performed, and the specimen was obtained by cutting the intercondylar notch in a box-shaped fashion using an osteotome, in order to preserve the femoral ACL insertion. The tibial plateau was cut with an oscillating saw as planned during the TKR. Menisci were removed from the tibial plateau leaving only the most peripheral tibial attachments, as the standard technique for TKR in order to have the optimal visualization of tibial plateau during the cut. TKR was performed in a standard fashion, and specimen harvesting had no consequence on the TKR technical execution or other intraoperative choices such as implant choice, degree of constrain, soft tissue balancing, blood loss, and surgical time.

The harvested specimen was kept at full knee extension position and fixed in 10% neutral-buffered formalin for 48 h to stabilize the tissue components. After washing in distilled water for 24 h, the sample was fully dehydrated using a graded series of ethanol (70, 80, 90, and 100%), by immersing in hexamethyldisilazane and air.

### Micro-Computed Tomography Analysis

The dry sample was scanned with the μCT system Skyscan 1176 (Bruker microCT, Kontich, Belgium) at a source voltage of 45 kV, a source current of 550 μA, with an aluminum filter of 0.2 mm, and an exposure of 185 ms. The nominal resolution was set at 17.5 μm. For the reconstruction in tomographic 2D sections, we used the dedicated NRecon software (version 1.6.10.4, Bruker micro-CT) applying corrections for specific misalignment, a low ring artifact reduction, and beam hardening. 3D model of the knee and its components (bone, cartilage, ACL, PCL, and menisci) was created using Avizo software (Thermo-Fisher Scientific). In order to distinguish the different knee joint components, we both divided the knee in volumes of interest and used global threshold depending on the different X-ray absorption of mineralized and soft tissues. The components were then virtually colored in the overall 3D model to enhance their differentiation.

## Ligaments and Menisci Appearance

**Figure 3:**
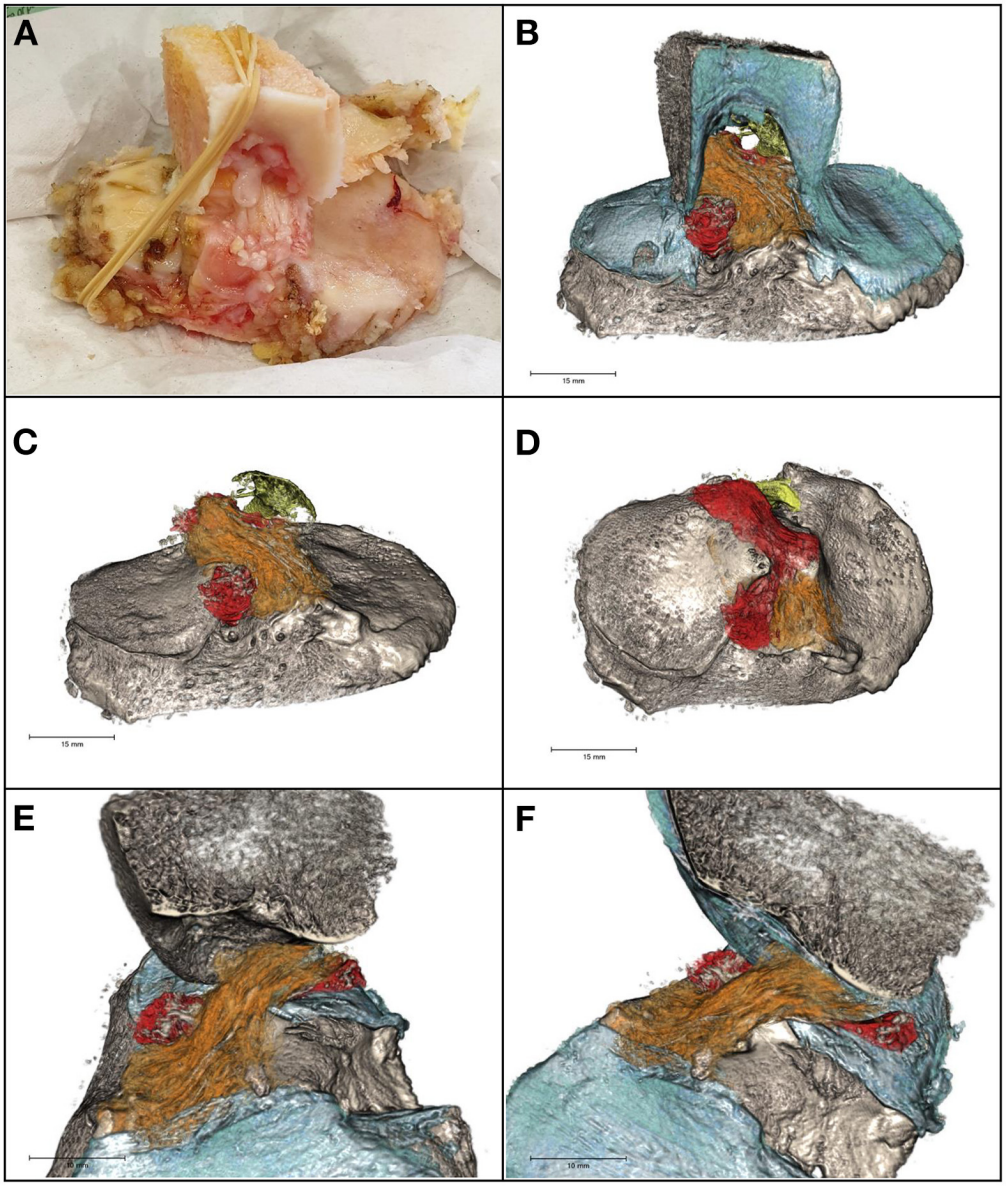
**(A)** The specimen. **(B)** 3D model of intercondylar notch, tibial plateau, cartilage (blue), ACL (orange) and PCL (yellow) tibial insertions, and lateral meniscus posterior and anterior horn insertions (red). Calcifications (gray) within the cruciate ligaments and the meniscal insertions supplemental fibers were linear in shape. **(C)** Antero-posterior view of ACL, PCL, and lateral meniscus tibial insertions. **(D)** Axial view of ACL, PCL, and lateral meniscus tibial insertions. **(E,F)** Sagittal view of the tibial plateau, ACL, and lateral meniscus tibial insertions.

The post-acquisition elaboration of the specimen allowed to build a 3D model of the tibial plateau, ACL, PCL tibial insertion, and lateral meniscus posterior and anterior horn insertions (Figure 3 and Supplementary Video 1). According to the views from coronal, axial, and sagittal planes, multiples intra-substance calcifications within the whole ACL structure and diameter were appreciated. Their shape was linear and sinuous, running parallel to ACL fibers. With the 3D reconstruction and by varying the view of inclination angle, it was possible to follow the multiple calcifications from the tibial insertion to the femoral origin, thus suggesting the shape and course of the ligament itself.

**Figure 4:**
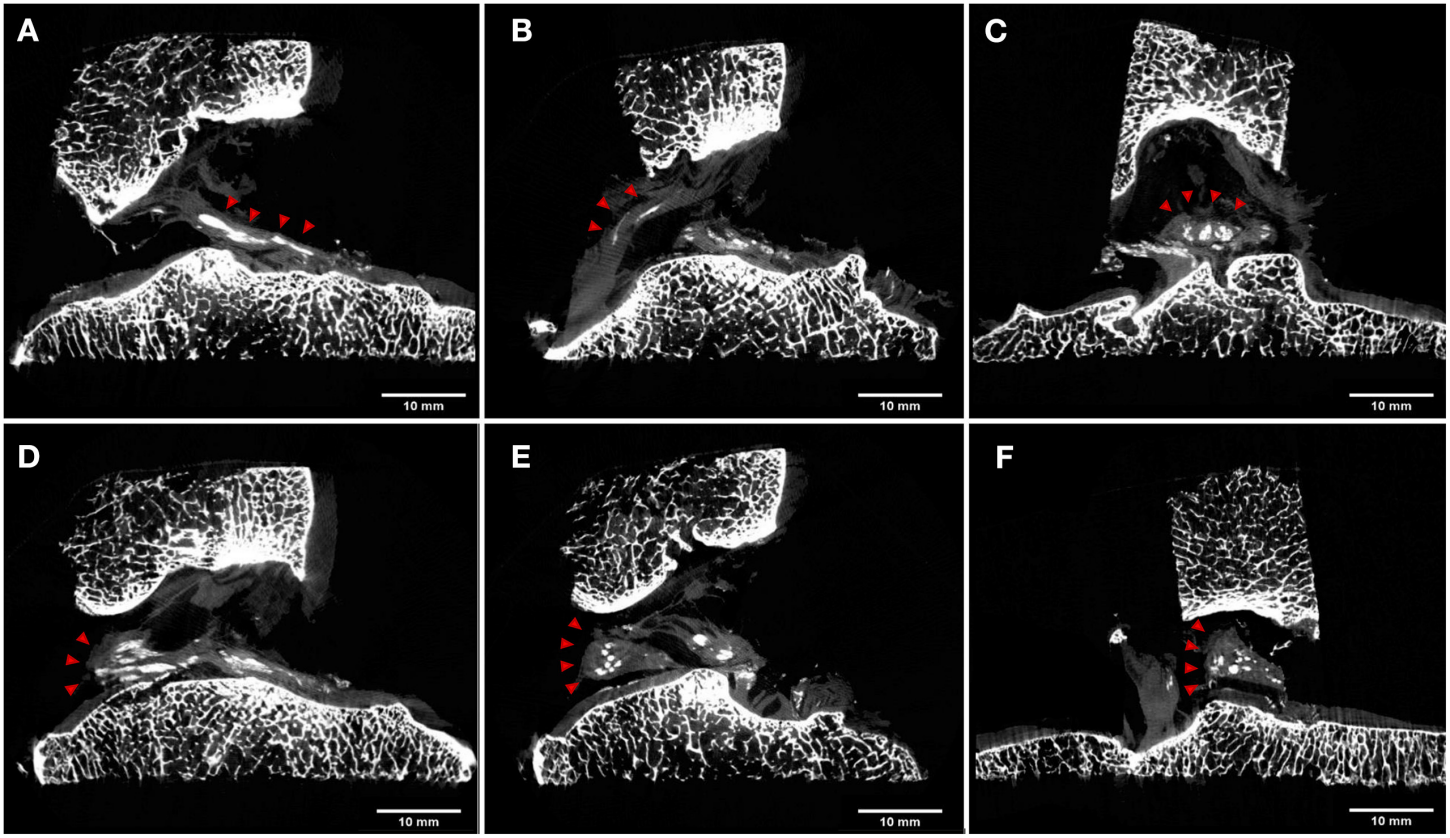
μCT cross sections. Calcifications are highlighted with red arrowheads. Sagittal views of ACL **(A)** and PCL **(B)** showed a linear pattern of the cruciate ligament calcifications. On the coronal plane, calcifications of ACL were characterized by an oval shape due to the virtual cut along their shortened diameter **(C)**. Sagittal views of lateral meniscus posterior horn insertion and flat menisco-tibial ligament **(D)** showed linear pattern calcifications, while the triangular meniscal remnant showed round or oval calcification both in the sagittal **(E)** and in the coronal **(F)** view.

Examining the specimen on the sagittal plane, it was appreciated the linear course of the calcifications along the ACL (Figure 4A). A similar pattern was present in the distal part of the PCL included in the specimen (Figure 4B). Differently, on the coronal plane centered on the tibial insertion (Figure 4C), it was possible to note the calcifications with an oval shape, due to the virtual cut along their shorted diameter.

A similar presentation was noted for the flat menisco-tibial ligament at the posterior horn insertions of the lateral meniscus included in the specimen where calcifications were linear on sagittal cuts (Figure 4D). Differently, calcifications within the triangular meniscal remnant were observed to be round or oval in shape in all planes (Figures 4E,F).

## Discussion

In this case report, we described a patient with primary knee OA and chondrocalcinosis who underwent total knee arthroplasty in whom an incidental postoperative diagnosis of ACL calcification was made using μCT scan.

The main finding of this case report was that μCT technology permits to obtain a 3D analysis of intra-articular calcium crystal deposition within ligament fibers, without the need to section the specimen into slice as performed in previous cadaveric studies to analyze the involvement of ACL in CPPD crystal deposition (15, 16). Abreu et al. and Dirim et al. analyzed ACL calcification with high-spatial-resolution radiography (Faxitron; HP; McMinnville, OR, United States) of the slices obtained from sectioning frozen knee specimens and confirmed CPPD crystal deposition using hematoxylin–eosin on histologic analysis; the authors reported the calcification of ACL, respectively, in two (50%) of four and in five (62.5%) of eight knee specimens with CPPD deposition (15, 16). On the other hand, standard X-ray and MRI seemed to reveal evidence of calcification in meniscal cartilage and hyaline cartilage, respectively, while they may not be helpful in the evaluation of calcification within ACL (11, 20). In a case report of 2016, Hayashi et al. described an incidental diagnosis of ACL calcification in a patient with chondrocalcinosis: While the CPPD crystal deposition within meniscal fibro cartilage was recognized on X-ray, the ACL calcification was not visualized on X-ray but only at CT of the knee (3). Even in the presented case, the calcification of ACL was visible not on X-ray but only on μCT examination. In a CT imaging *in vivo* study of 2015, Misra et al. reported the calcification of ACL in 10 of 24 knees with radiographic chondrocalcinosis, providing the potential utility of CT scan in the evaluation of intra-articular calcium crystal deposition (17).

The 3D analysis provided by μCT scan technology in the current case report showed a linear and sinuous pattern of the ACL calcification, with the shape running parallel to the ligamentous fibers. A similar pattern was reported in the studies of Abreu et al. (16) and Dirim et al. (15), in which the authors described the calcifications linear in shape and elongated in the plane of the fibers of the ligament (Figure 4).

Moreover, in the current case report, a linear pattern of calcification within the PCL fibers was observed as well on micro-CT imaging (Figure 4B); in the cadaveric studies of Dirim et al. (15), all five knees with ACL calcification presented an associated PCL calcification, characterized by a linear shape very similar to that described in the current case report.

The calcifications in the lateral meniscal remnant in the current case report were seen to be punctate and oval (Figures 4E,F). A similar pattern was reported by previous cadaveric studies, in which meniscal calcifications were described as round or oval in shape and aggregated in clusters (15, 16). Differently, a calcification pattern similar to ligamentous structures such as ACL and PCL was observed in the menisco-tibial ligament at the level of lateral meniscus posterior horn, however with a different fiber orientation compared to cruciate ligament calcifications (Figure 4D). In authors' opinion, this finding could be explained because these calcifications developed within the supplemental fibers of the meniscal root attachment, which have an elongated linear shape (22).

In conclusion, the implication of the presented case and the few studies that the authors founded in the literature suggested that the involvement of ACL in the CPPD deposition disease is frequent, but the conventional X-ray and MRI could not be helpful in its identification. μCT scan enables the detection of calcium crystal in deeper intra-articular structure like ACL and represents an important tool to better understand the morphology of calcifications without the potential artifacts of classic histology. Moreover, μCT scan allows us to investigate in deep the shape and course of calcifications within the ligament fibers from multiple spatial view orientations and in a 3D manner. Further studies describing more cases from other patients are needed to confirm the potential of the μCT scan in the analysis of the knee intra-articular structure.

## Data Availability Statement

The raw data supporting the conclusions of this article will be made available by the authors, without undue reservation.

## Ethics Statement

Ethical review and approval was not required for the study on human participants in accordance with the local legislation and institutional requirements. The patients/participants provided their written informed consent to participate in this study. Written informed consent was obtained from the individual(s) for the publication of any potentially identifiable images or data included in this article.

## Author Contributions

AG, GD, and AP wrote the first draft of the article. AG and SZ performed the surgery. AG, SZ, and AP interpreted the results. AP designed and performed the μCT analysis. AG, GD, SZ, MF, and AP critically reviewed the drafts. All authors contributed to the article and approved the submitted version.

## Conflict of Interest

The authors declare that the research was conducted in the absence of any commercial or financial relationships that could be construed as a potential conflict of interest.

## Publisher's Note

All claims expressed in this article are solely those of the authors and do not necessarily represent those of their affiliated organizations, or those of the publisher, the editors and the reviewers. Any product that may be evaluated in this article, or claim that may be made by its manufacturer, is not guaranteed or endorsed by the publisher.

## Supplementary Material

Supplementary Video 1 μCT 3D model of the tibial plateau, ACL, PCL tibial insertion, and lateral meniscus posterior and anterior horn insertions.
